# ^18^F-Fluciclovine–Avid Axillary Lymph Nodes After COVID-19 Vaccination on PET/CT for Suspected Recurrence of Prostate Cancer

**DOI:** 10.2967/jnmt.121.263001

**Published:** 2022-03

**Authors:** Justin G. Peacock, Elisabeth A. Banks, Nathan McWhorter

**Affiliations:** 1 Department of Radiology, Uniformed Services University, Bethesda, Maryland;; 2 Department of Radiology, San Antonio Military Medical Center, San Antonio, Texas; and; 3 College of Natural Sciences, University of Texas at Austin, Austin, Texas

**Keywords:** PET/CT, COVID-19 vaccine, ^18^F-fluciclovine, axillary lymph nodes, inflammation, prostate cancer

## Abstract

Abnormally increased ^18^F-FDG avidity of axillary lymph nodes has become a frequent diagnostic dilemma on PET/CT in the current climate of global vaccinations directed against severe acute respiratory syndrome coronavirus 2. This avidity is due to the inflammatory response evoked by vaccines and the nonspecific nature of ^18^F-FDG uptake, which is increased in both malignant and inflammatory processes. Similarly, ^18^F-fluciclovine, an amino acid analog indicated for the assessment of biochemical recurrence of prostate cancer, may also demonstrate nonspecific inflammatory uptake. We report a case of ^18^F-fluciclovine PET/CT obtained for concern about prostate cancer. In this case, isolated avid lymph nodes were seen in the left axilla. A screening questionnaire revealed that the patient had recently received the second dose of the Pfizer-BioNTech coronavirus disease 2019 vaccine in his left shoulder, and hence, the uptake was determined to be reactive.

Abnormal axillary lymph node avidity associated with vaccination was first reported in 2003 on ^18^F-FDG PET/CT images of healthy individuals who had received the killed influenza vaccine in a study assessing lymphocyte activation (*1*). Since then, similar findings have been reported from numerous other vaccinations (*2*). Such findings are due to the inflammatory response elicited by vaccines and the resulting upregulation of glucose transporters by activated immune cells (*3*). With the widespread implementation of vaccinations in response to the coronavirus disease 2019 (COVID-19) pandemic, there has been a marked increase in this phenomenon, resulting in significant diagnostic challenges with ^18^F-FDG PET/CT (*2*).

## CASE REPORT

**FIGURE 1.**
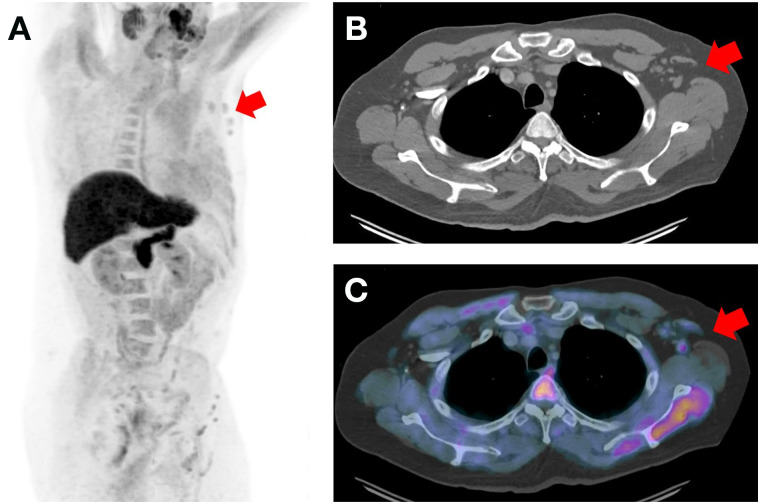
(A) ^18^F-fluciclovine PET maximum-intensity projection revealed 4 foci of unexpected avidity in left axilla (arrow). Remainder of uptake was physiologic. (B and C) Axial CT (B) and axial ^18^F-fluciclovine PET/CT (C) images show avidity localizing 4 mildly enlarged but morphologically normal lymph nodes (arrows). Remaining images (not shown) confirmed no other sites of potentially pathologic radiotracer avidity.

A 65-y-old man underwent surgery for prostate cancer. Subsequent pathologic evaluation revealed a Gleason score of 7 (3 + 4) with evidence of perineural invasion and 1 of 5 local lymph nodes positive for spread. There was no seminal vesicle invasion or extracapsular extension, and the surgical margins were negative. The overall stage was IVA (pT2, N1, M0). At 4 mo after surgery, there was a persistent detectable prostate-specific antigen level of 1.0 ng/mL, and ^18^F-fluciclovine (Axumin; Blue Earth Diagnostics) PET/CT was performed for further assessment. The examination revealed 4 foci of avidity (SUV_max_, 3.2) in the left axilla localizing to mildly enlarged but morphologically normal lymph nodes (Fig. 1). The remainder of the uptake was physiologic, with no other sites of pathologic radiotracer avidity. A review of the intake questionnaire showed that the patient had received the Pfizer-BioNTech COVID-19 vaccine in his left shoulder 17 d previously, and hence, the uptake was determined to be reactive. Subsequent clinical follow-up revealed an undetectable prostate-specific antigen level at 6 mo and again at 9 mo, confirming the benign nature of the uptake and the absence of residual malignancy.

## DISCUSSION

Although ^18^F-FDG is by far the most frequently used radiotracer in PET/CT, several additional radiotracers are in clinical use, including ^18^F-fluciclovine for imaging in men with suspected prostate cancer recurrence. ^18^F-fluciclovine is an amino acid analog and is taken up by prostate cancer (*4*). However, like ^18^F-FDG, ^18^F-fluciclovine can show increased uptake in inflammatory processes due to uptake by white blood cells. Given this fact, false-positive uptake has been reported in nonmalignant entities such as pneumonia, lymphadenitis, and ring worm infection (4*,*^5^). As with ^18^F-FDG, ^18^F-fluciclovine uptake by inflammatory processes tends to be mild compared with that by malignancy and most commonly has an intensity less than that in normal bone marrow (as in this case, with the L3 vertebra having an SUV_mean_ of 3.5). Hence, it is to be expected that, as with ^18^F-FDG, low-grade avidity by axillary nodes will be a potential finding on ^18^F-fluciclovine PET/CT after COVID-19 vaccination.

Recently, several prostate-specific membrane antigen PET tracers have received approval for use in the United States as an additional means for detecting prostate cancer. Despite their excellent performance, these, too, can demonstrate nonspecific uptake by inflammatory diseases such as pneumonia, and hence, it is likely only a matter of time before a case of COVID-19 vaccination–related uptake in the axillary lymph nodes is reported with these agents (*6*).

## CONCLUSION

The case underlines the importance of correlating examination findings with disease pathophysiology, radiotracer mechanism of action, and clinical history to optimize the accuracy of PET/CT interpretation.
